# Elucidating the dynamics and impact of the gut microbiome on maternal nutritional status during pregnancy, effect on pregnancy outcomes and infant health in rural Pakistan: study protocol for a prospective, longitudinal observational study

**DOI:** 10.1136/bmjopen-2023-081629

**Published:** 2024-08-12

**Authors:** Yaqub Wasan, Jo-Anna B Baxter, Carolyn Spiegel-Feld, Kehkashan Begum, Arjumand Rizvi, Junaid Iqbal, Jessie Hulst, Robert Bandsma, Shazeen Suleman, Sajid Soofi, John Parkinson, Zulfiqar Ahmed Bhutta

**Affiliations:** 1 Centre of Excellence in Women and Child Health, Aga Khan University, Karachi, Sindh, Pakistan; 2 Centre for Global Child Health, Hospital for Sick Children, Toronto, Ontario, Canada; 3 Department of Nutritional Sciences, University of Toronto, Toronto, Ontario, Canada; 4 Program in Molecular Medicine, Hospital for Sick Children, Toronto, Ontario, Canada; 5 Division of Gastroenterology, Hepatology and Nutrition, The Hospital for Sick Children, Toronto, Ontario, Canada; 6 Department of Nutritional Sciences and Department of Pediatrics, Temerty Faculty of Medicine, University of Toronto, Toronto, Ontario, Canada; 7 Department of Pediatrics, and Global Health Faculty Fellow, Centre for Innovation in Global Health, Stanford University, Stanford, California, USA; 8 Department of Pediatrics, Temerty Faculty of Medicine, University of Toronto, Toronto, Ontario, Canada; 9 Department of Biochemistry and Department of Molecular Genetics, University of Toronto, Toronto, Ontario, Canada; 10 Institute for Global Health and Development and Centre of Excellence in Women and Child Health, The Aga Khan University, Karachi, Sindh, Pakistan; 11 Department of Nutritional Sciences and Dalla Lana School of Public Health, University of Toronto, Toronto, Ontario, Canada

**Keywords:** Nutrition, PUBLIC HEALTH, EPIDEMIOLOGIC STUDIES, Observational Study, Body Mass Index

## Abstract

**Abstract:**

**Introduction:**

Undernutrition during pregnancy is linked to adverse pregnancy and birth outcomes and has downstream effects on the growth and development of children. The gut microbiome has a profound influence on the nutritional status of the host. This phenomenon is understudied in settings with a high prevalence of undernutrition, and further investigation is warranted to better understand such interactions.

**Methods and analysis:**

This is a prospective, longitudinal observational study to investigate the relationship between prokaryotic and eukaryotic microbes in the gut and their association with maternal body mass index (BMI), gestational weight gain, and birth and infant outcomes among young mothers (17–24 years) in Matiari District, Pakistan. We aim to enrol 400 pregnant women with low and normal BMIs at the time of recruitment (<16 weeks of gestation). To determine the weight gain during pregnancy, maternal weight is measured in the first and third trimesters. Gut microbiome dynamics (bacterial and eukaryotic) will be assessed using 16S and 18S rDNA surveys applied to the maternal stool samples. Birth outcomes include birth weight, small for gestational age, large for gestational age, preterm birth and mortality. Infant growth and nutritional parameters include WHO z-scores for weight, length and head circumference at birth through infancy. To determine the impact of the maternal microbiome, including exposure to pathogens and parasites on the development of the infant microbiome, we will analyse maternal and infant microbiome composition, micronutrients in serum using metallomics (eg, zinc, magnesium and selenium) and macronutrients in the stool. Metatranscriptomics metabolomics and markers of inflammation will be selectively deployed on stool samples to see the variations in dietary intake and maternal nutritional status. We will also use animal models to explore the bacterial and eukaryotic components of the microbiome.

**Ethics and dissemination:**

The study is approved by the National Bioethics Committee (NBC) in Pakistan, the Ethics Review Committee (ERC) at Aga Khan University and the Research Ethics Board (REB) at the Hospital for Sick Children, and findings will be published in peer-reviewed journals.

**Trial registration number:**

NCT05108675.

**STRENGTHS AND LIMITATIONS OF THIS STUDY**

- The study targets the high fertility age group (17–24) with almost half cohort consisting of low body mass index mothers, potentially with an additional risk of adverse pregnancy outcomes, providing an opportunity to comprehend the systematic understanding of the role of microbiota in several pregnancy, birth and infant outcomes.
- Study investigates both prokaryotic and eukaryotic dynamics of the gut microbiome for in-depth mechanistic insights in a highly malnourished population where contextual evidence is rare.
- Longitudinal design and data collection on a range of exposure indicators and biochemical analysis would enable us to evaluate the association of gut dynamics with several physiological and environmental factors.
- The study follows the Strengthening the Reporting of Observational Studies in Epidemiology guidelines; however, we expect controlling for all confounding variables may not be possible.
- Focusing on young women, 17–24 years of age, the findings may not be generalisable to younger or older demographics.

## Introduction

 Undernutrition during pregnancy is associated with an increased risk of poor birth outcomes and intrauterine growth restriction of the fetus^1 2^ and globally is a leading cause of death in under-5 children^3^ as well as maternal morbidity and mortality.^4 5^ Moreover, women with low body mass index (BMI) are at increased risk for adverse pregnancy and birth outcomes.^6 7^ Undernutrition before or during pregnancy may also have a long-term impact on the offspring.^8 9^ Among women in low-income and middle-income countries, several micronutrient deficiencies often coexist due to insufficient dietary intake.^1^ Pre-existing micronutrient deficiencies may be exacerbated during pregnancy as a result of the increased metabolic requirements.^10^ In particular, adolescent pregnancies are associated with an increased risk of stillbirths and neonatal deaths, an increased risk of preterm birth and low birth weight.^11^,^13^ In Pakistan, The National Nutritional Survey (2018) revealed the majority of women of reproductive age had some form of micronutrient deficiency, for example, 42.7% were anaemic. Nationwide, 40.2% of children under 5 were stunted, and 17.7% had severe wasting.^14^ Within the current study area, a previous multicentre study showed stillbirths and neonatal mortality rates in district Matiari^15^ were higher than the national rates.^16^

The intestinal microbiome has emerged as a key factor affecting nutritional status, with impaired maturation contributing to undernutrition.^17^,^22^ During pregnancy, dramatic changes to the gut microbiota occur, with a decrease in individual (alpha) diversity but an increase in population (beta) diversity.^23^ Relative to the first trimester, microbiota in the third trimester exhibit higher abundances of *Proteobacteria*, typically associated with obesity in humans, and when transplanted to mice, result in increased adiposity and insulin insensitivity. Such adaptations may increase energy extraction from the diet to support pregnancy,^24^ raising interest in dietary supplements to improve pregnancy outcomes.^25^

Maternal environmental enteric dysfunction (EED) during pregnancy is shown to adversely impact birth outcomes.^26 27^ Cumulative pathogen exposures in children confer a high risk for poor growth^28 29^ and EED.^30^,^33^ EED is thought to be triggered by dysbiosis,^34^,^36^ initiated by nutrient deficiencies, antibiotic treatment and/or pathogen exposure. This may further exacerbate pathogen colonisation, impair development of the mucosal immune system and disrupt, by as yet unknown mechanisms, metabolic processes that supply nutrients and energy for normal growth.^17^ Variability in microbial abundance and taxa in infancy and second year of life in association with exposure to different interventions and environment (rural and urban) in Pakistani children also warrants in-depth analysis including metagenomics and metabolomics to identify microbiota-directed interventions to address malnutrition.^34^

Thus, this study aims to systematically investigate how shifts in the gut microbial community impact nutritional status during pregnancy in young women. Leveraging whole microbiome RNASeq (metatranscriptomics) and metabolomics, our overall aim is to further develop mechanistic insights into the relationships between host socioenvironmental factors, nutritional status and microbiome dynamics during pregnancy, and how they contribute to birth outcomes and infant growth during the first year of life. Study findings may help devise microbiota-informed solutions to improve the nutritional status of mothers during pregnancy and improve birth outcomes and infant health, well-being and survival.

## Objectives

The primary aim of this study is to assess if alterations of the microbiota in the maternal gut (dysbiosis) are associated with maternal gestational weight gain and to determine the association between maternal microbiome dysbiosis during pregnancy and birth outcomes (ie, birth weight, preterm births, small for gestational age (SGA), large for gestational age (LGA)), infant growth, nutritional status and health status in the first year of life. Several secondary outcomes are integrated to better understand the influence of maternal factors such as dietary intake, maternal BMI and exposure to pathogens on the gut health and microbiome of infants. Finally, this study will explore how socioeconomic factors, including gender, poverty, exclusion and empowerment influence the health of a mother’s microbiome. Details are provided in online supplemental table 1.

### Study hypothesis

The study hypothesises that the alterations of the microbiota in the maternal gut (dysbiosis) exacerbated by nutritional status or pathogen exposure during pregnancy, impact weight gain during pregnancy because of impaired nutrient absorption, leading to corresponding negative birth outcomes.

## Methods

### Design

This study employs a prospective, longitudinal observational design. The design is best considered to study incidence, causes and prognosis. The approach allows investigators to measure the strength of the association of multiple exposures to an outcome and vice versa. Prospective data collection on exposures and potential confounders helps measure the change in outcomes with the level of exposure—increasing the generalisability of the results.^37^ Thus, longitudinal design and data collection on a range of exposure indicators and biochemical analysis in chronological order would enable us to investigate the causal link and strength of association of gut dynamics with several physiological and environmental factors and how they contribute to several maternal and infant health-related outcomes.

### Study setting and population

The study is being conducted in a rural district, Matiari, within Sindh province in Pakistan. Matiari is 200 km away from Karachi and includes more than 1400 villages and a population of about 800 000. The Aga Khan University (AKU) has a well-established community and health system liaison, basic demographic surveillance and field centres for research. This district is representative of typical conditions in Pakistan, and there is a close working relationship with the community, civic society leaders and public health departments. The study aims to collect blood and stool samples from pregnancy to 1-year post partum, to monitor the dynamic relationships between microbiome community structure and function with gut health and host nutritional status.

At the core of this study are two complementary cohorts of young women, one in Matiari, Pakistan, and one in Toronto, Canada. Here, we focus on the Pakistani cohort. We will recruit young, married women, including newlyweds, 17–24 years of age, living in Matiari District, Pakistan. We focused on this younger demographic due to our lack of knowledge on the microbiome of young women, and their increased vulnerability to undernutrition; two in every five young women (15–24) living in Matiari are underweight,^38^ many exhibit suboptimal dietary diversity^39^ and more than 90% experience at least one form of micronutrient deficiency.^40^ The cohort based in Toronto is focused on immigrant and refugee populations and will be described elsewhere.

### Study status

The first participant was enrolled on 25 November 2021. Data collection for the study is expected to be completed in 2024.

### Sample size

We aim to recruit 400 young women 17–24 years of age into two groups based on their BMI at timing of pregnancy identification: Group 1 will include those with a ‘normal’ BMI (ie, BMI between 18.5 and 25 kg/m^2^) and group 2 will include those with an underweight BMI (ie, <18.5 kg/m^2^), as per WHO’s guideline definitions.^41^ The adequacy of the sample size was verified using the ‘pwr’ package (V.1.2–2) in R (V.3.6.1). Calculations were based on the correlation between α-diversity (Shannon index) and weight gain during pregnancy. Assuming 400 participants are recruited into the study, and a type I error rate of 0.05, there will be 80% power to detect a correlation coefficient (r)>0.14. This is conventionally considered a small effect size.^42^ Thus, we expect to be powered to reveal a significant association between weight gain during pregnancy and microbial diversity.

### Participant identification

To identify the potential study participants, we leveraged the network that we had established in a previous trial, the Matiari emPowerment and Preconception Supplementation Trial.^43 44^ Using existing participant lists, women and families are initially approached by phone to get information on early pregnancies. Volunteers from within local villages and primary healthcare facilities are also encouraged to report new pregnancies. Verbal consent is taken from the identified pregnant women to share their pregnancy status with the study team for further eligibility screening. Research field staff also perform random checks of households and villages and meet with lady health workers and volunteers in the field to get information on pregnancies.

### Eligibility screening

Pregnancy identification data are assembled at the field office on a daily basis. The study team contacts the pregnant women for eligibility screening at their homes. The study staff obtains verbal consent to take the potential participant’s height and weight to determine the anthropometric eligibility criteria (BMI<25 kg/m^2^) and to conduct a pregnancy test to confirm pregnancy. Women 17–24 years of age who are in good general health, do not report frequent hospitalisation or recurrent episodes of any disease, without known chronic diseases, that is, hypertension, tuberculosis, diabetes mellitus, HIV, hepatitis B and C and cancer or any other, and <16 weeks of gestation are invited to take part in the study. To participate, women are asked to provide written consent and agree to comply with the study procedure. Those with a BMI≥25 kg/m^2^ at the time of recruitment and/or, participating in another nutritional trial, and/or report taking antibiotics in the last 3 months, and/or screening for potential signs of COVID-19 are not eligible for study participation. Given the potential for within-household dietary and microbial similarities, only one participant per household is enrolled in the study.

### Participant recruitment

Pregnant women who meet the eligibility criteria are formally invited for study participation following written informed consent. Informed consent is administrated by pursuing AKU‘s well-established protocols for research ethics compliance. Participants are also explained the option to sign an additional section of the consent form for future genetic testing of biospecimens. Participants are allowed to discuss the consent form with their families, before agreeing to participate in the study.

### Data and specimen collection

All data collection is completed by trained female data collectors at the participant’s home. This includes verbal data collection, anthropometric assessments, and blood and stool sample collection. Data collection at birth, which is collected either at a health facility or at home, depends on where the mother is available soon after delivery. Study research staff aim to complete birth anthropometrics within 72 hours postdelivery. Time points of data collection, biospecimen and anthropometric assessments are provided in online supplemental table 2. For all structured interviews with the study participants, study personnel use tablet-based Research Electronic Data Capture (REDCap) applications to guide data collection customised to the visit.^45 46^

#### Demographics

Household and personal demographics captured in this study include information regarding the participant’s age, gender, sex, occupation, the language spoken in the home, religion, income, number of people this income supports, education, housing, defecation, handwashing, reproductive history and marital status. Data collection scales are adapted from the Pakistan Demographic and Health Survey.^16^

#### Assessment of nutritional status

We use multiple approaches to measure the nutritional status of mothers and infants. This multimethod approach of direct and proxy indicators allows for a comprehensive evaluation of dietary intake, access to food, infant feeding practices and risks, growth patterns, and overall nutritional health. These methods include anthropometric measures, 24-hour food recall, minimum dietary diversity score for women (MDD-W), Household Food Insecurity Assessment Scale, infant and young child feeding practices and NutricheQ questionnaire. Details on these are provided below. Additionally, blood analyses include haemoglobin (HB), mean cell volume (MCV), C reactive protein (CRP), ferritin and metallomics analysis to investigate micronutrients (eg, zinc, magnesium, selenium (see the ‘Blood analysis’ section)).

#### Anthropometry

Maternal height and weight are measured using a digital floor scale (Seca 813, Seca, Hamburg, Germany) and stadiometer (Seca 213) based on which BMI is calculated (weight in kg/ height in m^2^); mid-upper arm circumference (MUAC) is taken using a measuring tape (Seca 201). Triceps skinfold thickness (SFT) is measured using a skinfold calliper (Holtain CRYMYCH, UK). We use Seca scales for infant anthropometry, which includes measurement of weight (Seca 354), length (Seca 417), MUAC (Seca 201) and head circumference (Seca 212). All measurements are collected in duplicate, by two study personnel, using standardised procedures, as adopted from the anthropometric data collection tools used in the INTERGROWTH-21st Study.^47^ The average of acceptable paired measures is used in subsequent analyses.

#### 24-hour food recall

To link the microbiome to nutritional status and nutritional intake, with a focus on calories and macronutrients, an interactive semiquantitative, 24-hour paper-based dietary recall^48^ is administered by the research staff at the participants’ homes.

#### MDD-W and household food insecurity

The MDD-W is a population-level indicator for dietary diversity for women aged 15–49, based on 10 food groups.^49^ The MDD-W reflects what a participant has eaten over the previous 24 hours, and participants are asked at the end of the questionnaire whether this reflects their diet over the previous 3 months. The research team will calculate the MDD-W from the dietary recalls completed at baseline, 30–34 weeks postconception and at 12 months. Food insecurity is assessed through the Household Food Insecurity Access Scale (HFIAS).^50^

#### Infant feeding

WHO-developed tools are used to assess infant feeding practices.^51^ At the 12-month visit, research staff administered the NutricheQ questionnaire, a tool designed for toddlers aged 1–3 years of age, with a focus on markers for inadequate or excessive intake and dietary imbalances.^52^ Two food insecurity questions, which assess maternal and infant annual food insecurity, are also included.^50^

#### Maternal empowerment

An empowerment questionnaire is deployed to collect data about self-efficacy using the Generalised Self-Efficacy scale.^53^ Perceived social support is measured using the Multidimensional Scale of Perceived Social Support.^54^ Perceived parental stress is measured using the Perceived Stress Scale-10.^55^

#### Birth history and pregnancy outcomes

Birth and labour history questions administered at the post-conception visit (24–72 hours) gather additional information on the mode of delivery, gestational age, newborn anthropometrics (weight, length, head circumference), placental insufficiency and antibiotic use, among other birth characteristics.

#### Health and medicine use assessment

Morbidity assessment captures mortality, morbidity and medication usage of mother and infant at several time points provided in online supplemental table 2 .

#### Blood sample collection

Certified paramedics are trained by AKU’s faculty and senior management of the Nutrition Research Laboratory (NRL) on blood collection using AKU’s Standard Operating Procedures. Venous blood specimens are collected from participating mothers (5 mL) and infants (3 mL) and distributed into two types of vacutainers. 0.6 mL of blood transferred to an SST tube (Yellow cap BD vacutainer: BD, PL6 7BP, UK) for ferritin and CRP analysis while the rest will be transferred to an EDTA tube (Purple cap BD vacutainer: BD, 1 Becton Drive, Franklin Lakes, NJ 07417 USA) for HB and MCV analysis, as well as for future storage. These tubes will be transported to the field-based lab in a Coleman portable freezer maintained at 2°C–8°C. The extracted serum will then be flash-frozen and stored at −80°C until the point of analysis.

#### Stool sample collection

Participating mothers are provided with sterile stool containers a day before collection to provide freshly passed stool samples to field staff while infants’ samples are collected in diapers and transferred to containers by the field team. If the stool is mixed with urine, collections are rescheduled. Collected samples are transported to the field-based lab in a Coleman portable freezer maintained at 2–8°C. At the lab, the sample is aliquoted in four cryovials and stored at −80°C until further processing.

## Patient and public involvement

The study participants have not been involved in the design, implementation, or analysis, or dissemination plans. However, before the initiation of study, we conducted meetings with community gatekeepers to orient them about the overall goal and the activities of the study.

## Data management

All tablets are synchronised daily to upload data from the REDCap data platform to a secure, web-based server hosted at the AKU campus. The tablets include built-in logic and range checks to ensure data quality. Paper-based study forms for the 24-hour food recall are checked by a study monitor for consistency and completeness. Dual entry of paper-based data is performed to reduce data entry errors. Data entry screens are developed using Visual FoxPro software (Microsoft). Deidentified data are stored in a password-protected database.

## Outcome measures

### Primary outcome measures

To determine the weight gain, maternal weight is measured in the first and third trimesters. Gut microbiome dynamics/dysbiosis (bacterial and eukaryotic) will be assessed using 16S and 18S rDNA surveys applied to the maternal stool samples. Birth outcomes include birth weight, SGA, LGA, preterm births (birth before 37 weeks of gestation), mortality and morbidity.

### Infant health outcomes

Infant growth and nutritional parameters include WHO z-scores for weight, length and head circumference at birth and during infancy till age 12 months.^56^ Beyond this, we will also monitor infant morbidity, mortality, care seeking, hospitalisation, antibiotic use, feeding practices and gut dynamics to establish an association between maternal gut dysbiosis and several birth and infant health outcomes.

### Secondary outcome measures

To determine the impact of the maternal microbiome including exposure to pathogens and parasites on the development of the infant microbiome with downstream consequences for nutrient uptake, we will analyse maternal and infant microbiome composition, micronutrients in the serum (using metallomics eg, zinc, magnesium and selenium) and macronutrients in the stool. Maternal stool markers of intestinal mass, inflammation (calprotectin, lipocalin-2 and claudin-15), and gut permeability and microbiome dynamics will be used to examine the association of intestinal inflammation with microbiome’s exposure to pathogens and parasites. We will integrate maternal clinical information (ie, morbidity, care seeking, medication use and hospitalisation) and anthropometric factors with microbiome data to reveal key modulators (microbial taxa and metabolites) of dietary intake during pregnancy and the postpartum period. Household food insecurity and dietary diversity scores will be generated to link the maternal microbiome to dietary intake, with a focus on calories and macronutrients. Additionally, previous studies have shown that dietary factors have a significant impact on the developing infant gut microbiome. These include timing and duration of breast feeding, supplementation or replacement with infant formulas, and timing and composition of solid food. For example, relative to breastmilk, feeding with soymilk formulas can alter clinically important metabolic pathways.^57^ Early introduction to solids (<3 months) has further been associated with increased microbial diversity and higher levels of short-chain fatty acids at 12 months.^58^ Thus, we will analyse the impact of feeding practices on several infant health outcomes including gut dynamics in relation to the duration of exclusive breast feeding, the timing and introduction of formula and animal milk (alone and in combination with breastmilk), and the introduction and timing of complementary foods.

### Exploratory outcomes

For the exploratory outcomes, metatranscriptomics, metabolomics and markers of inflammation will be selectively deployed on the stool samples to see the variations in dietary intake and maternal nutritional status. We will also use animal models to explore the bacterial and eukaryotic components of the microbiome. Details on outcome measures are provided in online supplemental table 1. Potential confounders are outlined under the ‘Strengths and limitations’ section.

## Stool analysis

Maternal and infant stool samples from all time points undergo DNA extraction at the NRL at AKU. Maternal stool RNA extraction will be completed for selected participants (100 with the highest BMI and 100 with the lowest BMI at the time of enrolment) from stool collected at the two pregnancy visits. Stool samples of these participants will also be analysed for inflammatory markers (table 1). The stool samples and extracted DNA and RNA samples will be then batch shipped to the Hospital for Sick Children, Toronto, Canada to complete downstream analyses, including sequencing, metabolomics and biobanking.

**Table 1:** Stool assays, methods, instruments and processing laboratories

Stool analyte	Amount of stool (mg)	Assay method	Instrument	Processing lab
Calprotectin	100	Sandwich Immunoassay	Liaison immune analyzer, Diasorin.	NRL, AKU, Pakistan
Lipocalin-2	100	Sandwich Immunoassay	Epoch 2 microplate reader, Biotek.	NRL, AKU, Pakistan
Claudin-15	300	Competitive Enzyme Immunoassay	Epoch 2 microplate reader, Biotek.	NRL, AKU, Pakistan
RNA extraction	250	Column Extraction Technology	ZymoBIOMICS RNA Miniprep Kit (R2001)	NRL, AKU, Pakistan
DNA extraction	200	Column Extraction Technology	ZymoBIOMICS DNA Miniprep Kit (D4300)	NRL, AKU, Pakistan
Stool archiving for shipment	2000	–	ThermoScientific TSX-Series −80°C Ultralow temperature freezer	NRL, AKU, Pakistan
Stool metabolomics*	100	Microbiome Metabolism Assay	LC-MS	TMIC, Alberta, Canada

* short-chain fatty acids (SCFAs), amino acids, intermediates in glycolysis and nucleotide metabolism.

AKUAga Khan University’sLC-MSLiquid chromatography mass spectrometryNRLNutrition Research LaboratoryTMICThe Metabolomics Innovation Centre

## Blood analysis

Blood samples will be analysed at AKU for HB, MCV, ferritin and CRP concentration, with additional aliquots shipped to SickKids for further analysis (table 2). The Metabolomics Innovation Centre (TMIC) will conduct metallomics analysis using the TMIC metallomics platform to investigate micronutrients (eg, zinc, magnesium and selenium).

**Table 2:** Blood assays, methods and processing laboratories

Blood analyte	Amount of blood/serum sample (μL)	Assay method	Instrument used	Processing lab
Haemoglobin+mean cell volume	500	Photometric assay	Sysmex p100 hematology analyzer	Matiari Research Laboratory, AKU, Pakistan
Ferritin	200	Immunoturbidimetric assay	CobasC311Analyzer, Roche diagnostics	NRL, AKU, Pakistan
C reactive protein	200	Immunoturbidimetric assay	CobasC311Analyzer, Roche diagnostics	NRL, AKU, Pakistan
Serum archiving for shipment	1000	–	ThermoScientific TSX-Series −80°C Ultralow temperature freezer	NRL, AKU, Pakistan
Serum metabolomics study	50	Metallomics assay	ICP-MS	TMIC, Alberta, Canada

AKUAga Khan UniversityICP-MSInductively Coupled Plasma-Mass SpectrometryNRLNutrition Research LaboratoryTMICThe Metabolomics Innovation Centre

### Profiling microbial community structure

Microbial communities will be analysed through 16S and 18S rDNA surveys using established methods that target the V4 region of the 16S rRNA gene to capture bacterial taxa^59^,^61^ and the V4V5 region of the 18S rRNA gene to capture eukaryotic taxa.^62^ DNA library preps include error-correcting barcodes^63^ for multiplexing of samples. Sequencing will be performed to generate ~50 000 2×150 bp paired end reads per sample. To define taxonomic diversity, species profiles from 16S and 18S rDNA data will be clustered to identify differences in community structure across samples. We will use the QIIME2 platform,^64^ MOTHUR,^65^ multivariate approaches such as permutation multivariate analysis of variance (ie, PERMANOVA-S a method that can associate microbiome changes with outcome measures while accounting for confounders).^66^ Differences between groups in microbiome community structure will be tested by analysis of similarities and co-occurrence analysis.^67^ To better define bacterial pathogen burden, we apply TaqMan array card technology for the simultaneous detection of 19 common enteropathogens.^68^

### Profiling microbial community function

After total RNA extraction and rRNA depletion (RiboZero Gold Kit, Illumina, San Diego, California, USA or equivalent), libraries will be constructed and Illumina-based sequencing will be performed to generate ~30 million 2×150 bp paired-end reads per sample (our rarefaction analyses have previously shown such sequencing depth is sufficient to identify the vast majority of species and enzymes present in the samples).^69^ Reads will be processed for quality and contaminants using the MetaPro pipeline.^70^ Reads will be assembled using SPAdes^71^ and subsequently annotated with taxonomic and functional assignments. Expression will be normalised to Reads per Kilobase of transcript per million mapped reads. Annotations will be mapped onto biochemical pathways and complexes such as those defined by the Kyoto Encyclopaedia of Genes and Genomes.^72^ The output of these analyses will be readouts of microbial gene expression detailing biochemical activities as well as the taxa responsible.

## Statistical analysis

Normally distributed continuous data will be shown as a mean and SD, and median and IQR will be calculated for non-normally distributed data. Categorical data will be presented using proportions.

For the primary outcomes, maternal gut bacteria and eukaryotic profiles will be used to calculate Bray-Curtis dissimilarity metrics between individual samples which is leveraged in principal co-ordinate analyses to determine the extent samples collected at the first or third trimester, exhibiting similar gestational weight gains, cocluster. PERMANOVA tests will assess the degree of overlap between samples exhibiting low gestational weight gain versus samples exhibiting high gestational weight gain. Next, we will attempt to correlate changes in the alpha diversity (as measured by the Shannon and Simpson indices) of the gut microbiome samples between the first and third trimester, with gestational weight gain. To examine the influence of individual taxa on gestational weight gain, we will perform bivariate analyses (Pearson, Spearman). The Benjamini-Hochberg procedure will be applied to correct p values while controlling for false-discovery rates.

To complement these analyses, we will also undertake an integrative modelling strategy based on the Similarity Network Fusion Framework^73^ to analyse the contribution of each variable (clinical, microbiome, socioeconomic and gender-related, see online supplemental table 1 on gestational weight gain. This allows the integration of all available datasets to uncover their global substructures that can be associated with gestational weight gain. In an alternative approach, we will also employ random forests to identify combinations of variables that correlate with gestational weight gain.

For the rest of the outcomes, followed by general linear models that is, PERMANOVA and the Bray-Curtis dissimilarity metric. DESeq2, a method for differential analysis of count data, will be applied to investigate both associations of specific taxa with the clinical variables, together with the strength of those associations. These analyses reveal which clinical variables (including exposure to pathogens and parasites) correlate with the maternal microbiome from a taxonomic perspective. Additionally, microbiome structural and functional profiles will be generated from the difference in (1) taxonomic abundances; (2) gene expression and (3) metabolite concentrations, between the first and third trimesters. Profile differences will be used in the PERMANOVA and DESeq2 approaches as described above to identify associations between clinical variables and changes in microbiome structure and function. Throughout these analyses, we will include potential confounders such as household and individual demographics, dietary intake, medication use, the impact of flooding and time of sample collection as outlined in the ‘Strengths and limitations’ section, as covariates where appropriate. Where consented, patient DNA data offer additional opportunities to integrate host genetics into these analyses (see online supplemental material).

## Discussion

Nutritional status during pregnancy plays an important role in maternal health and birth outcomes.^74^,^76^ Maternal undernutrition during pregnancy can lead to fetal growth restriction, which increases the risk of neonatal deaths and childhood stunting by 2 years of age.^3^ In Pakistan, large-scale surveys and cohort studies have suggested multifaceted undernutrition and adverse pregnancy and birth outcomes.^14^,^16^ Data from a cohort of young women (15–24 years) living in rural Pakistan revealed that more than 90% live with a minimum of one micronutrient deficiency^40^ and nearly 40% were underweight.^38^ Dietary intake was limited to fewer types of foods, mainly staples.^39^

The gut microbiome can have a profound influence on a host’s nutritional status, yet few studies of the dynamics between nutritional status and the gut microbiome during pregnancy have been conducted. Further, a few studies focusing on child undernutrition have revealed a key role for gut microbiota.^17^,^22^ In particular, dysbiosis, or the loss of diversity/beneficial microbes and the gain of pathobionts, has emerged as a major factor in the development of undernutrition. To date, most studies of the gut microbiome have focused on bacterial components, typically neglecting the contribution of eukaryotic microbiota. Despite the fact that many such eukaryotes include parasites, such as *Giardia*, *Cryptosporidium* and *Entamoeba*, each representing a significant burden on global healthcare with considerable implications for gut health.^77^,^79^ Interestingly, not all parasitic infections cause disease; instead, many infections remain asymptomatic with disease emerging as a consequence of interactions between the eukaryotic and bacterial microbiome and the host immune system.^80 81^ With the emergence of new marker gene technology, based on the 18S/5S/28S locus, there is now the opportunity to profile eukaryotic communities and examine their impact within the context of the gut microbiome.

Thus, this study will inform the relationships between host nutritional status and microbiome dynamics during pregnancy, and how they contribute to gestational weight gain during pregnancy, in addition to several other pregnancy and infant health-related outcomes. Understanding the role of the microbiome on maternal health, birth outcomes, infant health as well as the influence of enteric eukaryotic microbes, such as parasites, on the bacterial microbiome and host nutrition offers great potential in the identification of modifiable factors to improve health and nutrition outcomes.

To help establish causal relationships between microbiome dynamics, pathogen exposure and nutritional status during pregnancy and to examine whether manipulation of the microbiome can improve nutritional status, future work is expected to leverage stool samples collected here, in faecal microbiome transplant studies using animal models. The study findings may be instrumental in devising microbiota-informed strategies to improve the nutritional and pregnancy outcomes of mothers and the health and survival of infants living in similar settings. The finding can have the potential to inform microbiome-focused dietary guidelines for expecting mothers for improved nutritional outcomes and subsequent effects on pregnancy and infant outcomes. The incorporation of socioeconomic, food insecurity, gender and empowerment-related factors could potentially inform the multisectoral interventions to help improve the general health and well-being of mothers living in resource-limited settings.

## Strengths and limitations

The study targets the high fertility age group (17–24) with almost half of the cohort consisting of mothers with a low BMI, potentially with an additional risk of adverse pregnancy outcomes, providing an opportunity to comprehend the systematic understanding of the role of microbiota in several pregnancy, birth and infant outcomes. The study investigates both prokaryotic and eukaryotic dynamics of the gut microbiome for in-depth mechanistic insights in a highly malnourished population where contextual evidence is rare. The longitudinal design and data collection on a range of exposure indicators and biochemical analysis would enable the analysis to evaluate the association of gut dynamics with several physiological and environmental factors. The study follows the Strengthening the Reporting of Observational Studies in Epidemiology guidelines;^82^ however, we expect controlling for all biases and confounding variables may not be possible. For example, verbal data are collected through interviews while some follow-ups collect 3–6 months of recall data on morbidity, medication use and care-seeking which may impact the reliability of reporting mothers. However, to increase the accuracy of reporting, participants are encouraged to keep a record of medicines, that is, prescriptions and reports. We have developed a pictorial list of medicines to improve mothers’ recall. Of particular note, the study population experienced an unprecedented flooding event in 2022 which disrupted daily life and dietary patterns. Further, the flooding event is also expected to increase exposure of the study population to additional pathogens, with corresponding impacts on microbiome dynamics. We anticipate performing a subgroup analysis to investigate if exposure to flooding had a significant impact on microbial dynamics. We acknowledge that using BMI as a proxy for nutritional status and dividing up the cohort may be oversimplifying the data as it will not give insight into the body composition or functional status of the pregnant women. To provide more insight into this, we also included other measures of nutritional status such as MUAC and SFT, as well as dietary habits assessment and blood analysis of nutritional markers. Due to restricted hours of operation relative to the passing of stool by infants, we expect that there may be heterogeneity in the amount of time between the passing of stool and collection by the field team. At the same time, during sample collection, we ensure that the specimen temperature is maintained until it reaches the field-based laboratory. During analysis, we may find that we identify no significant differences between microbial diversity or composition in relation to our primary or secondary outcomes. Such findings would elevate the importance of the metatranscriptomic analyses to deliver more mechanistic investigations. It is possible that RNA quality and yields from stool is poor. In such events, we will revert to performing whole microbiome DNA (which is more stable than RNA) sequencing (metagenomics) which also has the capacity to deliver functional insights. Finally, we acknowledge that by focusing on young women, 17–24 years of age, the findings may not be generalisable to younger or older demographics. At the same time, studies of the microbiome within this age group are lacking and hence this study is aimed at directly addressing this knowledge gap to deliver a wealth of information to better serve the healthcare needs of this important demographic.

## Ethics

This study was approved by the National Bioethics Committee (NBC) in Pakistan (NBC Ref: No.4-87/NBC-700/21/820), the institutional Ethics Review Committee (ERC) at AKU (ERC No.2021-6085-17561) and the Research Ethics Board (REB) at the Hospital for Sick Children (SickKids; REB number: 1000076773).

## Dissemination

The study is registered with ClinicalTrials.gov Identifier: NCT05108675. Results will be published in open-access peer-reviewed journals.

## supplementary material

10.1136/bmjopen-2023-081629 online supplemental file 1

## References

[1] Ramakrishnan U, Grant F, Goldenberg T (2012). Effect of women’s nutrition before and during early pregnancy on maternal and infant outcomes: a systematic review. Paediatr Perinat Epidemiol.

[2] Uberos J, Jimenez-Montilla S, Machado-Casas I (2022). The association between restricted intra-uterine growth and inadequate postnatal nutrition in very-low-birth-weight infants and their neurodevelopmental outcomes: a 50-month follow-up study. Br J Nutr.

[3] Black RE, Victora CG, Walker SP (2013). Maternal and child undernutrition and overweight in low-income and middle-income countries. Lancet.

[4] Todd CS, Chowdhury Z, Mahmud Z (2019). Maternal nutrition intervention and maternal complications in 4 districts of Bangladesh: a nested cross-sectional study. PLoS Med.

[5] Tomkins A (2001). Nutrition and maternal morbidity and mortality. Br J Nutr.

[6] Katz J, Lee AC, Kozuki N (2013). Mortality risk in preterm and small-for-gestational-age infants in low-income and middle-income countries: a pooled country analysis. Lancet.

[7] Tang J, Zhu X, Chen Y (2021). Association of maternal pre-pregnancy low or increased body mass index with adverse pregnancy outcomes. Sci Rep.

[8] Odhiambo JF, Pankey CL, Ghnenis AB (2020). A review of maternal nutrition during pregnancy and impact on the offspring through development: evidence from animal models of over- and undernutrition. Int J Environ Res Public Health.

[9] Marshall NE, Abrams B, Barbour LA (2022). The importance of nutrition in pregnancy and lactation: lifelong consequences. Am J Obstet Gynecol.

[10] Berti C, Biesalski HK, Gärtner R (2011). Micronutrients in pregnancy: current knowledge and unresolved questions. Clin Nutr.

[11] Karaçam Z, Kizilca Çakaloz D, Demir R (2021). The impact of adolescent pregnancy on maternal and infant health in Turkey: systematic review and meta-analysis. J Gynecol Obstet Hum Reprod.

[12] Maheshwari MV, Khalid N, Patel PD (2022). Maternal and neonatal outcomes of adolescent pregnancy: a narrative review. Cureus.

[13] Marvin-Dowle K, Soltani H (2020). A comparison of neonatal outcomes between adolescent and adult mothers in developed countries: a systematic review and meta-analysis. Eur J Obstet Gynecol Reprod Biol X.

[14] UNICEF (2019). National Nutritional Survey 2018, Pakistan.

[15] Ahmed I, Ali SM, Amenga-Etego S (2018). Population-based rates, timing, and causes of maternal deaths, stillbirths, and neonatal deaths in south Asia and sub-Saharan Africa: a multi-country prospective cohort study. Lancet Glob Health.

[16] Studies NIoP (2018). Pakistan Demographic and Health Survey (PDHS) 2017–18.

[17] Blanton LV, Barratt MJ, Charbonneau MR (2016). Childhood undernutrition, the gut microbiota, and microbiota-directed therapeutics. Science.

[18] Barratt MJ, Ahmed T, Gordon JI (2022). Gut microbiome development and childhood undernutrition. *Cell Host & Microbe*.

[19] Chen RY, Mostafa I, Hibberd MC (2021). A microbiota-directed food intervention for undernourished children. N Engl J Med.

[20] Walter J (2024). Establishing microbiome causality to tackle malnutrition. Nat Microbiol.

[21] Chang H-W, Lee EM, Wang Y (2024). Prevotella copri and microbiota members mediate the beneficial effects of a therapeutic food for malnutrition. Nat Microbiol.

[22] Subramanian S, Huq S, Yatsunenko T (2014). Persistent gut microbiota immaturity in malnourished Bangladeshi children. Nat New Biol.

[23] Koren O, Goodrich JK, Cullender TC (2012). Host remodeling of the gut microbiome and metabolic changes during pregnancy. Cell.

[24] Astbury S, Mostyn A, Symonds ME (2015). Nutrient availability, the microbiome, and intestinal transport during pregnancy. Appl Physiol Nutr Metab.

[25] Bisanz JE, Enos MK, PrayGod G (2015). Microbiota at multiple body sites during pregnancy in a rural Tanzanian population and effects of moringa-supplemented probiotic yogurt. Appl Environ Microbiol.

[26] Lauer JM, Duggan CP, Ausman LM (2018). Biomarkers of maternal environmental enteric dysfunction are associated with shorter gestation and reduced length in newborn infants in Uganda. Am J Clin Nutr.

[27] Cowardin CA, Syed S, Iqbal N (2023). Environmental enteric dysfunction: gut and microbiota adaptation in pregnancy and infancy. *Nat Rev Gastroenterol Hepatol*.

[28] Islam MS, Zafar Ullah AN, Mainali S (2020). Determinants of stunting during the first 1,000 days of life in Bangladesh: a review. *Food Science & Nutrition*.

[29] Mutasa K, Tome J, Rukobo S (2022). Stunting status and exposure to infection and inflammation in early life shape antibacterial immune cell function among Zimbabwean children. Front Immunol.

[30] Semba RD, Trehan I, Li X (2017). Environmental enteric dysfunction is associated with carnitine deficiency and altered fatty acid oxidation. EBioMedicine.

[31] Crane RJ, Jones KDJ, Berkley JA (2015). Environmental enteric dysfunction: an overview. Food Nutr Bull.

[32] Jones KD, Thitiri J, Ngari M (2014). Childhood malnutrition: toward an understanding of infections, inflammation, and antimicrobials. Food Nutr Bull.

[33] Chen D, McKune SL, Singh N (2021). Campylobacter Colonization, environmental enteric dysfunction, stunting, and associated risk factors among young children in rural Ethiopia: a cross-sectional study from the Campylobacter Genomics and Environmental Enteric Dysfunction (CAGED) Project. Front Public Health.

[34] Levy M, Kolodziejczyk AA, Thaiss CA (2017). Dysbiosis and the immune system. Nat Rev Immunol.

[35] Petersen C, Round JL (2014). Defining dysbiosis and its influence on host immunity and disease. Cell Microbiol.

[36] Volin N, Thompson L, Cohen MK (2021). Maintaining and expanding the REFINE repository for food assistance research.

[37] Wang X, Kattan MW (2020). Cohort studies: design, analysis, and reporting. Chest.

[38] Baxter J-AB, Wasan Y, Hussain A (2023). Drivers of malnutrition among late adolescent and young women in rural Pakistan: a cross-sectional assessment of the MaPPS trial. BMJ Open.

[39] Baxter JB, Wasan Y, Islam M (2022). Dietary diversity and social determinants of nutrition among late adolescent girls in rural Pakistan. *Maternal & Child Nutrition*.

[40] Baxter J-AB, Wasan Y, Hussain A (2021). Characterizing micronutrient status and risk factors among late adolescent and young women in rural Pakistan: a cross-sectional assessment of the MaPPS trial. Nutrients.

[41] World Health Organization (1995). Physical Status: The Use of and Interpretation of Anthropometry, Report of a WHO Expert Committee.

[42] Cohen J (1988). Statistical Power Analysis for the Behavioral Sciences.

[43] Baxter J-AB, Wasan Y, Soofi SB (2018). Feasibility and effect of life skills building education and multiple micronutrient supplements versus the standard of care on anemia among non-pregnant adolescent and young Pakistani women (15-24years): a prospective, population-based cluster-randomized trial. Reprod Health.

[44] Baxter J-AB, Wasan Y, Soofi SB (2018). Effect of life skills building education and micronutrient supplements provided from preconception versus the standard of care on low birth weight births among adolescent and young Pakistani women (15-24years): a prospective, population-based cluster-randomized trial. *Reprod Health*.

[45] Harris PA, Taylor R, Minor BL (2019). The REDCap consortium: building an international community of software platform partners. J Biomed Inform.

[46] Harris PA, Taylor R, Thielke R (2009). Research electronic data capture (REDCap)--a metadata-driven methodology and workflow process for providing translational research informatics support. J Biomed Inform.

[47] Villar J, Cheikh Ismail L, Victora CG (2014). International fetal and newborn growth consortium for the 21st century (INTERGROWTH-21st). International standards for newborn weight, length, and head circumference by gestational age and sex: the newborn cross-sectional study of the INTERGROWTH-21st project. Lancet.

[48] Gibson R, Ferguson E (2008). An Interactive 24-Hour Recall for Assessing the Adequacy of Iron and Zinc Intakes in Developing Countries.

[49] Project I (2018). Data4Diets: Building Blocks for Diet-Related Food Security Analysis.

[50] Coates J, Swindale A, Bilinsky P (2007). Household Food Insecurity Access Scale (HFIAS) for Measurement of Household Food Access: Indicator Guide.

[51] World Health Organization (2010). Indicators for Assessing Infant and Young Child Feeding Practices: Part 2 Measurement.

[52] Morino GS, Cinelli G, Di Pietro I (2015). NutricheQ questionnaire assesses the risk of dietary imbalances in toddlers from 1 through 3 years of age. Food Nutr Res.

[53] Schwarzer R, Jerusalem M (2010). The general self-efficacy scale (GSE). Anxiety Stress Coping.

[54] Zimet GD, Dahlem NW, Zimet SG (1988). The multidimensional scale of perceived social support. J Pers Assess.

[55] Cohen S, Kamarck T, Mermelstein R (1983). A global measure of perceived stress. J Health Soc Behav.

[56] Perumal N, Shi J, Bassani D (2016). Length‐for‐age and weight‐for‐age*z*scores at birth using the world health organization growth standards versus the new INTERGROWTH 21^st^newborn size standards. FASEB J.

[57] Baumann-Dudenhoeffer AM, D’Souza AW, Tarr PI (2018). Infant diet and maternal gestational weight gain predict early metabolic maturation of gut microbiomes. Nat Med.

[58] Differding MK, Benjamin-Neelon SE, Hoyo C (2020). Timing of complementary feeding is associated with gut microbiota diversity and composition and short chain fatty acid concentrationsover the first year of life. BMC Microbiol.

[59] Coburn B, Wang PW, Diaz Caballero J (2015). Lung microbiota across age and disease stage in cystic fibrosis. Sci Rep.

[60] Copeland JK, Yuan L, Layeghifard M (2015). Seasonal community succession of the phyllosphere microbiome. Mol Plant Microbe Interact.

[61] Tyler AD, Knox N, Kabakchiev B (2013). Characterization of the gut-associated microbiome in inflammatory pouch complications following ileal pouch-anal anastomosis. PLoS ONE.

[62] Popovic A, Bourdon C, Wang PW (2018). Design and application of a novel two-amplicon approach for defining eukaryotic microbiota. Microbiome.

[63] Hamady M, Walker JJ, Harris JK (2008). Error-correcting barcoded primers for pyrosequencing hundreds of samples in multiplex. Nat Methods.

[64] Caporaso JG, Kuczynski J, Stombaugh J (2010). QIIME allows analysis of high-throughput community sequencing data. Nat Methods.

[65] Schloss PD, Westcott SL, Ryabin T (2009). Introducing mothur: open-source, platform-independent, community-supported software for describing and comparing microbial communities. Appl Environ Microbiol.

[66] Tang Z-Z, Chen G, Alekseyenko AV (2016). PERMANOVA-S: association test for microbial community composition that accommodates confounders and multiple distances. Bioinformatics.

[67] Weiss S, Van Treuren W, Lozupone C (2016). Correlation detection strategies in microbial data sets vary widely in sensitivity and precision. ISME J.

[68] Liu J, Gratz J, Amour C (2013). A laboratory-developed TaqMan array card for simultaneous detection of 19 enteropathogens. J Clin Microbiol.

[69] Xiong X, Bales ES, Ir D (2017). Perilipin-2 modulates dietary fat-induced microbial global gene expression profiles in the mouse intestine. Microbiome.

[70] Taj B, Adeolu M, Xiong X (2023). MetaPro: a scalable and reproducible data processing and analysis pipeline for metatranscriptomic investigation of microbial communities. Microbiome.

[71] The Metabolomics Innovation Centre (2019). Services.

[72] Kanehisa M, Goto S, Hattori M (2006). From genomics to chemical genomics: new developments in KEGG. Nucleic Acids Res.

[73] Wang B, Mezlini AM, Demir F (2014). Similarity network fusion for aggregating data types on a genomic scale. Nat Methods.

[74] Vaivada T, Gaffey MF, Das JK (2017). Evidence-based interventions for improvement of maternal and child nutrition in low-income settings: what’s new?. Curr Opin Clin Nutr Metab Care.

[75] Bhutta ZA, Das JK, Rizvi A (2013). Evidence-based interventions for improvement of maternal and child nutrition: what can be done and at what cost?. Lancet.

[76] Kabir MA, Rahman MM, Khan MN (2022). Maternal anemia and risk of adverse maternal health and birth outcomes in Bangladesh: a nationwide population-based survey. PLoS One.

[77] Van de Voorde M, Van Hecke K, Binnemans K (2018). Separation of samarium and europium by solvent extraction with an undiluted quaternary ammonium ionic liquid: towards high-purity medical samarium-153. RSC Adv.

[78] Angelici MC, Walochnik J, Calderaro A (2021). Free-living amoebae and other neglected protistan pathogens: health emergency signals. Eur J Protistol.

[79] Das R, Palit P, Haque MA (2023). Symptomatic and asymptomatic enteric protozoan parasitic infection and their association with subsequent growth parameters in under five children in South Asia and sub-Saharan Africa. PLoS Negl Trop Dis.

[80] Allain T, Fekete E, Sosnowski O (2021). High-fat diet increases the severity of Giardia infection in association with low-grade inflammation and gut microbiota dysbiosis. Sci Rep.

[81] Funkhouser-Jones LJ, Xu R, Wilke G (2023). Microbiota-produced indole metabolites disrupt mitochondrial function and inhibit cryptosporidium parvum growth. Cell Rep.

[82] von Elm E, Altman DG, Egger M (2007). Strengthening the reporting of observational studies in epidemiology (STROBE) statement: guidelines for reporting observational studies. BMJ.

