# Supplementary material for: Elucidating the dynamics and impact of the gut microbiome on maternal nutritional status during pregnancy, effect on pregnancy outcomes and infant health in rural Pakistan: study protocol for a prospective, longitudinal observational study
Source: BMJ Open. 2024 Aug 12;14(8):e081629. doi: 10.1136/bmjopen-2023-081629 (PMC11331926; doi:10.1136/bmjopen-2023-081629)
Supplement: Supplementary file 2 [file bmjopen-2023-081629.reviewer_comments.pdf]

## PEER REVIEW HISTORY

BMJ Open publishes all reviews undertaken for accepted manuscripts. Reviewers are asked to complete a checklist review form (**http://bmjopen.bmj.com/site/about/resources/checklist.pdf**) and are provided with free text boxes to elaborate on their assessment. These free text comments are reproduced below.

## ARTICLE DETAILS

| | |
|---|---|
| **TITLE (PROVISIONAL)** | Elucidating the Dynamics and Impact of the Gut Microbiome on Maternal Nutritional Status During Pregnancy, Effect on Pregnancy Outcomes, and Infant Health in Rural Pakistan: Study Protocol for a Prospective, Longitudinal Observational Study. |
| **AUTHORS** | Wasan, Yaqub; Baxter, Jo-Anna B.; Spiegel-Feld, Carolyn; Begum, Kehkashan; Rizvi, Arjumand; Iqbal, Junaid; Hulst, Jessie; Bandsma, Robert; Suleman, Shazeen; Soofi, Sajid; Parkinson, John; Bhutta, Zulfiqar |

## VERSION 1 – REVIEW

| | |
|---|---|
| **REVIEWER** | Iftikhar Alam<br>Bacha Khan University Charsadda, Bacha Khan University, Charsadda |
| **REVIEW RETURNED** | 23-Mar-2024 |

| | |
|---|---|
| **GENERAL COMMENTS** | Dear Editor,<br>Thank you for inviting me to review this very interesting study protocol. I want to congratulate the authors for the effort in writing this interesting paper. The manuscript is very well written and easy to follow and it would be interesting to expand such a study in a bigger one and collect data from the same babies also later on in life to follow the trajectory of their microbiota and brain development.<br>I have a few small comments to be considered for the revised manuscript in the following:<br>1. The title alludes to a causative influence of microbes on the Maternal Nutritional Status. I would suggest changing it to more accurately describe the possible data and conclusion of the current study? Also, I believe the title is also missing the useful outcome related to the newborn/bay etc? would be good it it is modified accordingly.<br>2. Do the authors have any exclusion criteria as well: Those with a previous history of three abortions, cervical alterations, major fetal anomaly, Mullerian anomalies, history of scalpel cervical conization, chronic corticosteroid use and detected or self-reported preexisting disease including hypertensive disorder, previous diagnosis of diabetes mellitus, renal disease, systemic lupus erythematosus or antiphospholipid syndrome, sickle cell anemia and HIV positive serology, are excluded from the study. Women taking medication or supplementation that could interfere in the outcome assessment, such as aspirin, calcium, fish oil, vitamin C, vitamin E or heparin, are also excluded<br>3. How will the authors identify their potential candidates for the proposed study? Through newspaper? Random home-to-home visits etc? if you have done it previously, please give some details?<br>4. Maybe I missed it, but could the data being correlated also on the only breast milk feeding or mixed feeding or only solid food feeding? |

| | |
|---|---|
| | It was clear to me that data on the solid food introduction were available but it is not so clear to me at which extend the babies could be classified in the groups, if it is possible at all. If not, could the authors comment on the importance of diet in this early life stage and on the microbiota and early development?.<br>5. To assess nutrition status, most frequently, the body mass index (BMI) is calculated using the pre-pregnancy weight, which has a fundamental role in determining the total amount of weight gain, monitoring gestational development and providing nutritional counseling. However, when information about pre-pregnancy weight is missing, various health systems recommend the charts of Atalah according to gestational week. Furthermore, in low or middle-income countries, it is common for women to seek antenatal care late, without information about the previous nutritional status. Moreover, BMI, as the only factor associated with maternal nutrition, is unable to provide all the answers that trigger an adverse condition in the pregnant woman and her offspring. This information needs to be associated with anthropometric data and sociodemographic conditions, involving diverse dietary habits that bring a wealth of knowledge to the decision-making process. A variety of methods have been developed for the use in the assessment of the nutrient status. Conceptually, they fall into two categories: static measurements, which include determination of nutrient levels in blood or tissues, and functional measurements, which include a wide range of tests to determine the adequacy of nutritional status to support the functions of subcellular constituents, cells, tissues, organs, biologic systems, or the whole body. In the assessment of nutritional status during pregnancy, static measurements include determination of overt signs of clinical deficiency; estimates of tissue stores; and determination of level of nutrients or metabolites in maternal and infant blood and in other body tissues and fluids. Functional measurements include determination of activity of nutrient-dependent enzymes or amounts of hormones, nutrient-dependent metabolic or structural changes, rates at which maternal and infant anthropometric measurements change, and indicators of the course and outcome of pregnancy, including maternal and infant health, gestation duration, birth weight, and infant neurobehavioral development. I would recommend the authors may also consider these measures in their nutritional assessment protocols (or comment).<br>6. Lastly, I have not found any discussion on the possibility of the infant microbiota assessment as possible indicator of the maternal nutritional/microbiota status indicator? Would be interesting if this is also included in the methods. |

| | |
|---|---|
| **REVIEWER** | Ghulam HUSSAIN<br>Government College University Faisalabad, Physiology |
| **REVIEW RETURNED** | 02-Apr-2024 |

| | |
|---|---|
| **GENERAL COMMENTS** | In the current study entitled "Elucidating the Dynamics and Impact of the Gut Microbiome on Maternal Nutritional Status During Pregnancy in Rural Pakistan: Study Protocol for a Prospective, Longitudinal Observational Study" submitted under the category "protocol," the authors have elaborated on all aspects in a very clear and understandable way. |

| | |
|---|---|
| **REVIEWER** | Ihsan Ullah<br>The Aga Khan University |
| **REVIEW RETURNED** | 19-Apr-2024 |

| | |
|---|---|
| **GENERAL COMMENTS** | Ensure robust data collection procedures to minimize bias and ensure the reliability of study findings. Delete the strengths and limitations below the Abstract. Consider additional strategies to address potential confounding factors, such as socioeconomic status and dietary intake. Explore opportunities for collaboration with local health authorities and community leaders to enhance participant recruitment and engagement. Continuously monitor and adjust study protocols as needed to address emerging challenges and optimize research outcomes. Overall, the manuscript provides a strong foundation for investigating the complex interplay between the gut microbiome and maternal nutritional status during pregnancy in rural Pakistan. Continued attention to methodological rigor and collaboration with relevant stakeholders will be critical for the success of the study. |

| | |
|---|---|
| **REVIEWER** | Shelina Bhamani<br>The Aga Khan University Hospital, Obstetric &Gynecology |
| **REVIEW RETURNED** | 22-Apr-2024 |

| | |
|---|---|
| **GENERAL COMMENTS** | 1. Please provide more description with the study design, why is it the most applicable one, how will it help investigation.<br>2. Mention how your research will have policy or practice implications<br>3. Try to pull references from studies beyond 2020 |

**VERSION 1 – AUTHOR RESPONSE**

Reviewer: 1
Dr. Iftikhar Alam
Comments to the Author:

Dear Editor,
Thank you for inviting me to review this very interesting study protocol. I want to congratulate the authors for the effort in writing this interesting paper. The manuscript is very well written and easy to follow and it would be interesting to expand such a study in a bigger one and collect data from the same babies also later on in life to follow the trajectory of their microbiota and brain development.
I have a few small comments to be considered for the revised manuscript in the following:

Thank you for your insightful suggestion to collect data from the same babies later on in life to follow the trajectory of their microbiota and brain development. We will be looking for such opportunities to further explore the downstream role of microbiota in achieving developmental outcomes.

1. The title alludes to a causative influence of microbes on the Maternal Nutritional Status. I would suggest changing it to more accurately describe the possible data and conclusion of the current study? Also, I believe the title is also missing the useful outcome related to the newborn/bay etc? would be good it is modified accordingly.

Thank you for identifying this, we greatly appreciate it. Given this is a protocol publication, at this time we don’t know about the conclusions and results, however, per your comment, we are revising the Title to reflect the study’s overarching aims which also look into the infant outcomes. Now this appears as

“*Elucidating the Dynamics and Impact of the Gut Microbiome on Maternal Nutritional Status during Pregnancy, Effect on Pregnancy Outcomes, and Infant Health in Rural Pakistan: Study Protocol for a Prospective, Longitudinal Observational Study”*.

2. Do the authors have any exclusion criteria as well: Those with a previous history of three abortions, cervical alterations, major fetal anomaly, Mullerian anomalies, history of scalpel cervical conization, chronic corticosteroid use and detected or self-reported preexisting disease including hypertensive disorder, previous diagnosis of diabetes mellitus, renal disease, systemic lupus erythematosus or antiphospholipid syndrome, sickle cell anemia and HIV positive serology, are excluded from the study. Women taking medication or supplementation that could interfere in the outcome assessment, such as aspirin, calcium, fish oil, vitamin C, vitamin E or heparin, are also excluded.

We are thankful for your thoughtful input. We agree that these diseases and conditions can substantially interfere with microbiota dynamics. We have considered such conditions in our exclusion criteria in the field.

The study exclusion criteria are briefly described under the **Secerning Eligibility** sub-heading, as under, text in black is added per your valuable suggestion.

*"Women 17-24 years of age who are in good general health, do not report frequent hospitalization or recurrent episodes of any disease, without known chronic diseases i.e., hypertension, tuberculosis, diabetes mellitus, HIV, hepatitis B & C, and cancer or any other, and <16 weeks of gestation are invited to take part in the study. To participate, women are asked to provide written consent and agree to comply with the study procedure. Those with a BMI ≥25 kg/$m^2$ at the time of recruitment and/or, participating in another nutritional trial, and/or report taking antibiotics in the last three months, and/or screen for potential signs of COVID-19 are not eligible for study participation. Only one participant per household is enrolled in the study, given the potential for within-household dietary and microbial similarities."*

To further operationalize the exclusion criteria in our staff training, by general good health, we meant those who did not report having frequent hospitalization or episodes of any disease/illness or physical disability were eligible for study participation. We did not specifically list all the names of conditions/diseases that you have mentioned here, because the majority of the study population is not well-educated and has access to a resource-limited health system. Therefore, there was a higher possibility that the participants might not be diagnosed or not be aware of these medical terminologies. We understand that frequent hospitalization or episodes of illness might be the signs of any disease/condition that could potentially interfere with the Microbiome dynamics.  That's why we kept these in the exclusion criteria. Additionally, the list of chronic diseases included known hypertension, hepatitis B and C, diabetes, HIV, cancers, renal disease, etc.

Regarding the use of medicine, someone who used antibiotics in the last 3 months at the time of recruitment was not eligible for participation. Additionally, we collected data on the use of medicine throughout the study, thus any confounding effects of medicine use will be considered at the time of analysis. This is mentioned in the analysis section.

3. How will the authors identify their potential candidates for the proposed study? Through newspaper? Random home-to-home visits etc? if you have done it previously, please give some details?

Thank you for this note. We have described this under the subheading <u>Participant Identification</u>. The text appears as below:

*To identify the potential study participants within the study area, we leveraged the network that we had established in a previous trial, the Matiari emPowerment and Preconception Supplementation (MaPPS) Trial [43, 44]. Using existing participant lists, women and families are initially approached by phone to get information on early pregnancies. Volunteers from within local villages and primary*

*healthcare facilities are also encouraged to report new pregnancies. Verbal consent is taken from the identified pregnant women to share their pregnancy status with the study team for further eligibility screening. Research field staff also perform random checks of households and villages and meet with lady health workers and volunteers in the field to get information on pregnancies.*

4. Maybe I missed it, but could the data being correlated also on the only breast milk feeding or mixed feeding or only solid food feeding? It was clear to me that data on the solid food introduction were available but it is not so clear to me at which extend the babies could be classified in the groups, if it is possible at all. If not, could the authors comment on the importance of diet in this early life stage and on the microbiota and early development?.

Yes, we collect data on infant feeding practices using the WHO standard IYCF questionnaire at three, six, and twelve months to record data on breastfeeding, complementary feeding, dietary diversity, and meal frequency. At the 12-month visit, research staff administers the NutricheQ questionnaire, a tool designed for toddlers aged 1 to 3 years of age, with a focus on markers for inadequate or excessive intake and dietary imbalances. We have mentioned this under the sub-heading Infant Feeding and Table 01.

Per your suggestion, the text is added under the subheading Secondary Outcome Measure, it appears as

*Additionally, previous studies have shown that dietary factors have a significant impact on the developing infant gut microbiome. These include timing and duration of breastfeeding, supplementation or replacement with infant formulas, and timing and composition of solid food. For example, relative to breastmilk, feeding with soymilk formulas can alter clinically important metabolic pathways [65] Early introduction to solids (< 3 months) has further been associated with increased microbial diversity and higher levels of short-chain fatty acids at 12 months [66]. Thus, we will analyze the association of feeding practices with several infant health outcomes including gut dynamics in relation to the duration of exclusive breastfeeding, the timing, and introduction of formula and animal milk (alone and in combination with breastmilk), and the introduction and timing of complementary foods.*

5. To assess nutrition status, most frequently, the body mass index (BMI) is calculated using the pre-pregnancy weight, which has a fundamental role in determining the total amount of weight gain, monitoring gestational development and providing nutritional counseling. However, when information about pre-pregnancy weight is missing, various health systems recommend the charts of Atalah according to gestational week. Furthermore, in low or middle-income countries, it is common for women to seek antenatal care late, without information about the previous nutritional status. Moreover, BMI, as the only factor associated with maternal nutrition, is unable to provide all the answers that trigger an adverse condition in the pregnant woman and her offspring. This information needs to be associated with anthropometric data and sociodemographic conditions, involving diverse dietary habits that bring a wealth of knowledge to the decision-making process. A variety of methods have been developed for the use in the assessment of the nutrient status. Conceptually, they fall into two categories: **static measurements**, which include determination of nutrient levels in blood or tissues, and functional measurements, which include a wide range of tests to determine the adequacy of nutritional status to support the functions of subcellular constituents, cells, tissues, organs, biologic systems, or the whole body. In the assessment of nutritional status during pregnancy, static measurements include determination of overt signs of clinical deficiency; estimates of tissue stores; and determination of level of nutrients or metabolites in maternal and infant blood and in other body tissues and fluids. **Functional measurements** include determination of activity of nutrient-dependent enzymes or amounts of hormones, nutrient-dependent metabolic or structural changes, rates at which maternal and infant anthropometric measurements change, and indicators of the course and outcome of pregnancy, including maternal and infant health, gestation duration, birth weight, and infant

neurobehavioral development. I would recommend the authors may also consider these measures in their nutritional assessment protocols (or comment).

Thank you for this insightful suggestion. Because women were recruited to the study in early pregnancy, it was not possible to include pre-pregnancy measures (i.e., due to study design). We also acknowledge that sole BMI does not provide a complete picture of nutritional status. However, we collect data on multiple proxy indicators to help understand the nutritional status of mothers and infants. We have added text in the manuscript (as appears below) and reorganized the section under the subheading Data and Specimen Collection, to better reflect the assessment of nutritional measures. We have also added a sub-heading under primary outcome measures to reflect the infant health-related primary outcome measures. Additionally, we have included a sentence in the limitation section about the use of BMI to describe maternal nutritional status (second paragraph below).

*Assessment of nutritional status*

*We use multiple approaches to measure the nutritional status of mothers and infants. This multi-method approach of direct and proxy indicators allows for a comprehensive evaluation of dietary intake, access to food, infant feeding practices and risks, growth patterns, and overall nutritional health. These methods include anthropometric measures, 24-hour food recall, minimum dietary diversity score for women (MDD-w), household food insecurity assessment scale(HHFIAS), infant and young child feeding practices (IYCF), and NutricheQ questionnaire. Details on these are provided below (see manuscript). Additionally, blood analyses include Hb, MCV, CRP, ferritin, and metallomics analysis to investigate micronutrients (e.g., zinc, magnesium, selenium (see blood analysis section)).*

Added text in the limitation section.

*We acknowledge that using BMI as a proxy for nutritional status and dividing up the cohort, may be oversimplifying the data as it won't give insight into the body composition or functional status of the pregnant women. To provide more insight into this, we also included other measures of nutritional status such as MUAC and skinfold thickness, as well as dietary habits assessment and blood analysis of nutritional markers*

6. Lastly, I have not found any discussion on the possibility of the infant microbiota assessment as possible indicator of the maternal nutritional/microbiota status indicator? Would be interesting if this is also included in the methods.

Thank you for pointing this out. We will do this analysis and we have briefly described this under the subheading Secondary Outcome Measures. Additionally, edits are made in the discussion part to reflect that this study analysis will also look into the impact of microbial dynamics on infant health outcomes. Moreover, Table 01 of the supplementary material contains details on the multiple maternal and infant outcome measures, including this one.

*"To determine the impact of the maternal, microbiome including exposure to pathogens and parasites on the development of the infant microbiome with downstream consequences for nutrient uptake we will analyze maternal and infant microbiome composition, micronutrients in the serum (using metallomics e.g., zinc, magnesium, and selenium), and macronutrients in the stool. Maternal stool markers of intestinal mass, inflammation (Calprotectin, Lipocalin-2, Claudin-15), and gut permeability and microbiome dynamics will be used to examine the association of intestinal inflammation with microbiome's exposure to pathogens and parasites. We will integrate maternal clinical information (i.e., morbidity, care seeking, medication use, and hospitalization) and anthropometric factors with microbiome data to reveal key modulators (microbial taxa and metabolites) of dietary intake during*

*pregnancy and the postpartum period. Household food insecurity and dietary diversity scores will be generated to link the maternal microbiome to dietary intake, with a focus on calories and macronutrients."*

Reviewer: 2
Dr. Ghulam HUSSAIN, Government College University Faisalabad, Government College University Faisalabad
Comments to the Author:
In the current study entitled "Elucidating the Dynamics and Impact of the Gut Microbiome on Maternal Nutritional Status During Pregnancy in Rural Pakistan: Study Protocol for a Prospective, Longitudinal Observational Study" submitted under the category "protocol," the authors have elaborated on all aspects in a very clear and understandable way.

Thank you so much for the time you spent reviewing this protocol. We greatly appreciate it.

Reviewer: 3
Dr. Ihsan Ullah, The Aga Khan University
Comments to the Author:
Ensure robust data collection procedures to minimize bias and ensure the reliability of study findings. Delete the strengths and limitations below the Abstract. Consider additional strategies to address potential confounding factors, such as socioeconomic status and dietary intake. Explore opportunities for collaboration with local health authorities and community leaders to enhance participant recruitment and engagement. Continuously monitor and adjust study protocols as needed to address emerging challenges and optimize research outcomes. Overall, the manuscript provides a strong foundation for investigating the complex interplay between the gut microbiome and maternal nutritional status during pregnancy in rural Pakistan. Continued attention to methodological rigor and collaboration with relevant stakeholders will be critical for the success of the study.

Thank you for your invaluable comments. I have broken down and placed each comment in the below table, to ensure that I have responded to all comments appropriately.

| Comments | Responses |
|---|---|
| 1. Ensure robust data collection procedures to minimize bias and ensure the reliability of study findings. | Thank you for your valuable input.<br><br>We adopted the internationally validated tool to measure the study outcomes. All tools were translated into the local language and pilot-tested to improve the context, understanding of the data collection team and participants, and eventual reliability. Standard Operating Procedures (SOPs) were developed for each assessment and data collection tool. Data collection applications have built-in quality checks for consistency and completeness. We have robust field and desk monitoring mechanisms to ensure data quality. |
| 2. Delete the strengths and limitations below the Abstract | We acknowledge your suggestion.<br><br>However, the organization of this draft follows the BMJ Open Journal's guidelines. We have described the strengths and limitations at the end of the study too. The same are presented in bullets as a pre-requisite of the Journal. We |

| | |
|---|---|
| | leave this to the editor to decide where to place this section. |
| 3. Consider additional strategies to address potential confounding factors, such as socioeconomic status and dietary intake. | We appreciate the insightful suggestion and recognize the importance of addressing these factors in our study. To mitigate the influence of socioeconomic status, we have collected data on participants' demographic characteristics, including household income, education level, occupation, etc., and plan to incorporate these variables into our statistical analyses as covariates. Additionally, we acknowledge the significance of dietary intake in shaping health outcomes and have implemented standardized dietary assessment tools to capture participants' dietary patterns and nutrient intake.<br><br>This has been described under the subheading Statistical Analysis. The text is also edited to reflect that we will consider the confounding effect of these factors. |
| 4. Explore opportunities for collaboration with local health authorities and community leaders to enhance participant recruitment and engagement. | Your insightful comment is appreciated. We have considered the suggested engagement strategies to achieve this task.<br><br>Additionally, within the manuscript document, we have edited the subheading **Pregnancy Identification** to<br><br>**Participant identification,** the text under it appears as:<br><br>*To identify the potential participants, we leveraged the network that we had established in a previous trial, the Matiari emPowerment and Preconception Supplementation (MaPPS) Trial [39, 40]. Using existing participant lists, women and families are initially approached by phone to get information on early pregnancies. Volunteers from within local villages and primary health care facilities area are also encouraged to report new pregnancies. Verbal consent is taken from the identified pregnant women to share their pregnancy status with the study team for further eligibility screening. Research field staff also perform random checks of households and villages and meet with lady health workers and volunteers in the field to get information on pregnancies.* |
| 5. Continuously monitor and adjust study protocols as needed to address emerging challenges and optimize research outcomes | Your insightful comment is appreciated. We will be looking into this and make adjustments with prior approval from ethics review boards if the need arises. |
| 6. Overall, the manuscript provides a strong foundation for investigating the complex interplay between the gut | Thank you for the note. We acknowledge helpful suggestions. We understand the importance of collaboration with relevant stakeholders for knowledge translation into |

| | |
|---|---|
| microbiome and maternal nutritional status during pregnancy in rural Pakistan. Continued attention to methodological rigor and collaboration with relevant stakeholders will be critical for the success of the study. | action. We will look for such opportunities in the future. |

Reviewer: 4
Dr. Shelina  Bhamani, The Aga Khan University Hospital
Comments to the Author:
1. Please provide more description with the study design, why is it the most applicable one, how will it help investigation.

Thank you for bringing this to our attention. We have edited the text that appears under the sub-heading **Study Design,** it appears as below:

*This study employs a prospective, longitudinal observational design. The design is best considered to study incidence, causes, and prognosis. The approach allows investigators to measure the strength of the association of multiple exposures to an outcome and vice versa. Prospective data collection on exposures and potential confounders helps measure the change in outcomes with the level of exposure – increasing the generalizability of the results [39]. Thus, longitudinal design and data collection on a range of exposure indicators and biochemical analysis in chronological order would enable us to investigate the causal link and strength of association of gut dynamics with several physiological and environmental factors and how they contribute to several maternal and infant health-related outcomes.*

2. Mention how your research will have policy or practice implications

Thank you for highlighting this valuable addition. A paragraph on this is added at the end of the discussion part. It appears as under:

*The study findings may be instrumental in devising microbiota-informed strategies to improve the nutritional and pregnancy outcomes of mothers and the health and survival of infants living in similar settings. The finding can have the potential to inform microbiome-focused dietary guidelines for expecting mothers for improved nutritional outcomes and subsequent effects on pregnancy and infant outcomes. The incorporation of socio-economic, food insecurity, gender, and empowerment-related factors could potentially inform the multisectoral interventions to help improve the general health and well-being of mothers living in resource-limited settings.*

3. Try to pull references from studies beyond 2020.
We appreciate you highlighting this. We have updated/supplemented the references throughout the introduction/background and discussion section. Newly added references are highlighted in the marked copy. However, in the methods and analysis section, we referred to either standard tools or methods of analysis which were developed before 2020 and are kept unchanged.

## VERSION 2 – REVIEW

| | |
|---|---|
| **REVIEWER** | Iftikhar Alam<br>Bacha Khan University Charsadda, Bacha Khan University, Charsadda |
| **REVIEW RETURNED** | 08-Jun-2024 |

| | |
|---|---|
| **GENERAL COMMENTS** | Dear Editor,<br>The authors have successfully revised the manuscript and I believe that the revised version has improved and may be considered for publication.<br>Regards<br>Iftikhar |

| | |
|---|---|
| **REVIEWER** | Shelina Bhamani<br>The Aga Khan University Hospital, Obstetric &Gynecology |
| **REVIEW RETURNED** | 25-Jun-2024 |

| | |
|---|---|
| **GENERAL COMMENTS** | Brilliant work in progress, looking forward to the results |
